# Obstetric and perinatal outcomes of singleton pregnancies conceived via assisted reproductive technology complicated by gestational diabetes mellitus: a prospective cohort study

**DOI:** 10.1186/s12884-018-2115-4

**Published:** 2018-12-14

**Authors:** Azam Kouhkan, Mohammad E. Khamseh, Reihaneh Pirjani, Ashraf Moini, Arezoo Arabipoor, Saman Maroufizadeh, Roya Hosseini, Hamid Reza Baradaran

**Affiliations:** 1 Endocrine Research Center, Institute of Endocrinology and Metabolism, Iran University of Medical Sciences (IUMS), Firouzeh St., South Vali- Asr Ave., Vali- Asr Sq., Tehran, 15937-16615 Iran; 2 Department of Endocrinology and Female Infertility, Reproductive Biomedicine Research Center, Royan Institute for Reproductive Biomedicine, ACECR, Number 12, East Hafez Avenue, Bani Hashem Street, Resalat Highway, Tehran, 16635-148 Iran; 3 Department of Gynecology and Obstetrics, Arash Women’s Hospital, Tehran University of Medical Sciences, Tehran, Iran; 4 Department of Epidemiology and Reproductive Health, Reproductive Epidemiology Research Center, Royan Institute for Reproductive Biomedicine, ACECR, Tehran, Iran; 5 Department of Andrology, Reproductive Biomedicine Research Center, Royan Institute for Reproductive Biomedicine, ACECR, Tehran, Iran; 6 Ageing Clinical & Experimental Research Team, Institute of Applied Health Sciences, University of Aberdeen, Aberdeen, Scotland AB25 2ZD UK

**Keywords:** Gestational diabetes mellitus, Assisted reproductive technology, Complications, Obstetric, Perinatal, Neonatal

## Abstract

**Background:**

Growing evidence indicates that the risk of obstetric and perinatal outcomes is higher in women with assisted reproductive technology (ART). However, there is little known about pregnancy related complications and co-morbidity in gestational diabetes mellitus (GDM) following singleton pregnancies achieved by ART in comparison with spontaneous conception (SC).

**Methods:**

Two hundred sixty singleton pregnant women conceived by ART and 314 pregnant women conceived by spontaneous conception (SC) were participated in this prospective cohort study. All participants were enrolled after GDM screening through one-step oral glucose tolerance test (OGTT) and then grouped into GDM and non-GDM groups. Women were followed for pregnancy outcomes including pregnancy-induced hypertension (PIH), preeclampsia, antepartum hemorrhage (APH), cesarean section (CS), preterm birth (PTB), intrauterine growth restriction (IUGR), being small or large for gestational age (SGA or LGA), macrosomia, low birth weight (LBW), respiratory distress, neonatal hypoglycemia, NICU admission and perinatal mortality from antenatal visits to delivery. Confounding factors were adjusted in logistic regression model in order to estimate adjusted odds ratios (aORs).

**Results:**

Among 260 ART and 314 SC, 135 and 152 women were GDM women, respectively. Higher maternal age and pre-gravid BMI, shorter duration of gestation and lower gestational weight gain were observed in GDM groups (ART-GDM and SC-GDM) compared to those of the SC group. ART-GDM group had a higher risk (95% confidence interval) of obstetric complications including PIH [aOR:7.04 (2.24–22.15)], preeclampsia [aOR:7.78 (1.62–37.47)], APH [aOR:3.46 (1.28–9.33)], emergency CS [aOR:2.64 (1.43–4.88)], and perinatal outcomes such as PTB [aOR:3.89 (1.51–10.10)], LBW [aOR:3.11 (1.04–9.30)] and NICU admission [aOR:4.36 (1.82–10.45)], as well as neonatal hypoglycemia [aOR: 4.91 (1.50–16.07)], compared to SC group. SC-GDM group showed a higher risk of PIH [aOR: 4.12 (1.31–12.89)], emergency CS [aOR: 2.01 (1.09–3.73] and LGA [aOR: 5.20 (1.07–25.20)], compared to SC group. Additionally, ART group had a higher risk of PIH [aOR: 3.46(1.02–11.68), preeclampsia 5.29 (1.03–27.09), and NICU admission [aOR: 2.53 (1.05–6.09)] compared to SC. Insulin requirement (41.8% vs. 25.7%) was significantly higher in ART-GDM group compared to SC-GDM group.

**Conclusion:**

The findings of this study suggest that GDM occurring after ART conception increases the risk of adverse obstetric and perinatal outcomes.

## Background

Gestational diabetes mellitus (GDM) is a common metabolic disorder of pregnancy, which has unfavorable and negative effects (e.g. occurrence of preeclampsia, macrosomia, low birth weight and preterm birth) on maternal and fetal health [1, 2]. Population studies have reported a GDM prevalence of 1–14% [3]. The Hyperglycemia and Adverse Pregnancy Outcome (HAPO) study indicated that higher levels of maternal glucose are related to increased risks of adverse pregnancy outcomes [4].

During the last decades, the number of pregnancies conceived using assisted reproductive technologies (ART) has increased globally [5]. A recent meta-analysis showed that infertility history and treatment are linked to increased risk of GDM, particularly in Asians [6]. Also, the wide range of GDM prevalence (11–40%) among those undergoing ART was reported [7–10]. Previous meta-analyses demonstrated that perinatal outcomes in pregnancies achieved by ART are poorer than those of pregnancies conceived naturally [11, 12]. Despite considerable improvement in ART protocols and laboratory techniques as well as enhanced practices such as elective single embryo transfer and frozen embryo transfer cycles, recent evidence indicated similar consequences [7, 13].

Though several investigations have studied maternal and perinatal outcomes of ART conception [8, 13–16] or outcomes of natural pregnancy complicated by GDM [17–20], to the best of our knowledge, no prospective cohort study has yet compared GDM -related outcomes between assisted conception and spontaneous pregnancies. A recent retrospective study done by Szymanska et al. [21], compared maternal and neonatal outcomes of GDM between women who underwent in vitro fertilization (IVF) and non-IVF women. However, consequences of ART pregnancies complicated by GDM are yet to be understood. Considering the increasing number of pregnancies achieved by ART, and increased awareness of GDM-related morbidities, it is of crucial importance to explore pregnancy-related complications in GDM pregnancies following ART. Therefore, the present study compared obstetric and perinatal outcomes of GDM between singleton pregnancies achieved by ART and those of spontaneous pregnancies, in order to have a clearer understanding of maternal and infant health under such conditions; the results of this study would be beneficial for policy makers with respect to healthcare interventions required for prevention and control of GDM among ART population.

## Materials and methods

This prospective observational cohort study was carried out in Royan Institute and maternity teaching hospital located in Tehran. The participants gave written informed consent for the data collection and ethical approval was granted by Institutional Review Boards and the Ethics Committees of Royan Institute, Tehran, Iran and Iran University of Medical Sciences, Tehran, Iran (date:2014-09-4, ethics code: IR.ACECR.ROYAN.REC.1393.2 and date:2015-09-4, ethics code: IR.IUMS.REC.1396.25469). This study was performed from November 2014 to January 2017.

The ART pregnancies were singleton pregnancies following IVF /intra-cytoplasmic sperm injection (IVF/ICSI) or ICSI cycles that treated at infertility clinic affiliated to Royan Institute. Women with spontaneous conception (SC) were those did not have a history of infertility (time to pregnancy < 1 years) and/or infertility treatments, and referred to Arash Women’s Hospital (affiliated to Tehran University of Medical Sciences, Tehran, Iran).

Singleton pregnant women aged 20–42 years, who conceived via ART or SC, were enrolled. All participants with a history of chronic diseases, multiple pregnancies, pre-pregnancy diabetes mellitus, and glucose intolerance as well as those who were receiving hypoglycemic agents [e.g. metformin for treatment of polycystic ovary syndrome (PCOS)], or corticosteroids as well as those with pregnancies with vanishing embryos or selective fetal reduction, were excluded from the study. Also, women with a history of infertility or infertility treatment were excluded from the SC group.

**Fig. 1:**
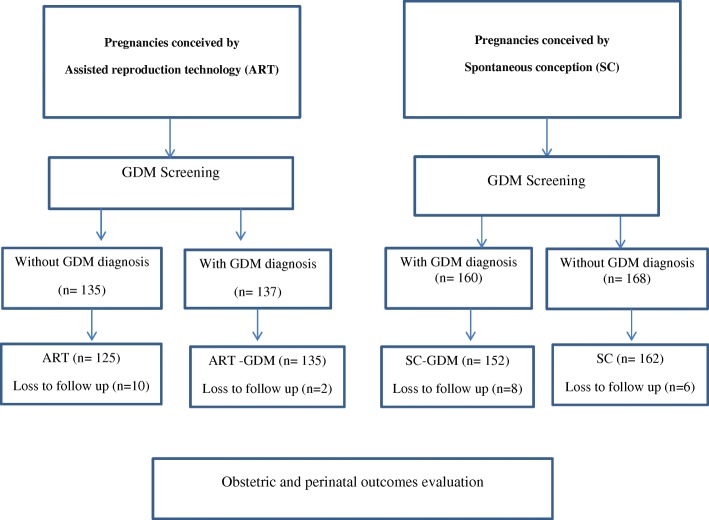
Flow chart of the study population

All women were evaluated for pre-existing diabetes by measurement of fasting blood sugar (FBS) during the first trimester of pregnancy and the results were recorded in hospital registry. Then, all participants were screened for GDM using one-step oral glucose tolerance test (OGTT done by oral administration of 75 g glucose) according to ADA/IAPDSG (American Diabetes Association/International Association of the Diabetes and Pregnancy Study Groups) criteria at 24–28 weeks of gestation except for high-risk women (high-risk subjects were those with a history of GDM and PCOS, age ≥ 35 years, and pre-gravid obesity). Both groups (ART and SC) were stratified into two groups (GDM and non-GDM) based on the results of OGTT. In the present study, 600 eligible singleton pregnant women conceived via ART or SC were enrolled at 24–28 weeks of gestation after screening for GDM. Twelve women from ART group and 14 women from SC group were excluded as they selected other centers for further prenatal care follow up or unwilling to continue the study. Finally, 574 singleton pregnant women were grouped into four groups namely, ART, ART with GDM (ART-GDM), SC with GDM (SC-GDM), and SC (Fig. 1).

Demographic and clinical data were obtained from patients’ medical records and hospital databases and through face-to-face interviews. All participants were followed for obstetric and perinatal outcomes of pregnancy. The data was obtained by telephone follow-up, clinical evaluations and checking hospital records by a trained physician from antenatal visits to 2 weeks after childbirth. All participants received prenatal care in Royan Institute and Arash Women’ hospital and the deliveries were performed at a university-based hospital for high-risk maternal-fetal medicine.

The following variables were included in the final analysis: maternal age; gravidity (primigravida: the first pregnancy); parity (nulliparous: no previous births); pre-pregnancy weight, height and pre-pregnancy BMI [pre-pregnancy weight (kg)/ (height (m)) ^2^]; gestational age at delivery (in weeks), type of GDM treatment (diet and/or insulin) during pregnancy, as well as gestational weight gain (GWG).

In both groups (ART and SC), GDM patients were referred to a dietitian for dietary counseling. After 2 weeks, blood sugar profile (BSP) in terms of FBS and postprandial (i.e. 2-h after breakfast, lunch and dinner) serum glucose levels, was determined. In case of FBS < 95 mg/dl and 2-h postprandial blood sugar levels < 120 mg/dl, dietary management alone was used. Patients with higher blood sugar levels were treated with subcutaneous injections of rapid-acting insulin and/or long-acting insulin. Blood sugar levels were monitored twice a week.

The following obstetric complications were assessed: pregnancy-induced hypertension (PIH; as defined by systolic blood pressure > 140 and/or diastolic blood pressure > 90 mmHg after 20 weeks of pregnancy), preeclampsia (pregnancy-induced hypertension associated with proteinuria ≥100 mg/dl as shown by urine analysis or ≥ 300 mg/24 h), antepartum hemorrhage (APH; any bleeding in the second or third trimesters due to placenta praevia, abruption, and uterine bleeding), and emergency cesarean (i.e. not planned cesarean).

Perinatal data including newborn’s birth weight, height and sex, as well as neonatal morbidity and mortality, were recorded at delivery. Apgar scores at 1 min and 5 min were retrieved from the delivery records. Perinatal outcomes including macrosomia (birth weight > 4000 g), low birth weight (LBW; birth weight < 2500 g), small for gestational age (SGA; birth weight < 10th percentile for a given gestational age), large for gestational age (LGA; birth weight > 90th percentile for a given gestational age), preterm birth (PTB; birth sooner than gestation week 37), neonatal hypoglycemia (blood glucose< 45 mg/dl), neonatal intensive care unit (NICU) admission, intrauterine growth restriction (IUGR; growth below the third percentile for gestational age), respiratory distress and perinatal mortality (combination of still birth and fetal and neonatal death) were also collected.

### Statistical analysis

Data analysis was done using IBM SPSS Statistics for Windows, Version 22.0 (IBM Crop., Armonk, NY, USA). On the basis of the incidence rate of PIH, preeclampsia and PTB as primary outcomes [9, 22, 23], the final sample size (with significance level *α* = 0.05 and power 1 − *β* = 0.8) was calculated and 130 women were enrolled in each group. In this study, continuous variables were expressed as mean ± standard deviation and categorical variables were expressed as number (percentage). Chi-square test and one-way ANOVA were used to compare baseline characteristics among groups where appropriate. To examine the effects of GDM and ART on pregnancy complications, logistic regression analysis was performed to estimate crude odds ratios (OR) and adjusted odds ratios (adjusted OR = aOR) with 95% confidence intervals (CI) and adjusted for maternal age, parity, and pre-pregnancy BMI. All statistical tests were two-sided and level of significance was set at 0.05.

## Results

After evaluation, 574 singleton pregnancies including 260 ART conception (135 subjects with and 125 without GDM diagnosis), and 314 spontaneous conception (152 subjects with and 162 without GDM diagnosis) were enrolled (Fig. 1). The baseline characteristics of the four groups are compared in Table 1. Women in SC group were significantly younger than the women of other groups (*p* < 0.05). Pre-gravid BMI was significantly higher in GDM groups (ART-GDM and SC-GDM) than non-GDM groups (ART and SC) (*P* < 0.01). Pre-gravid obesity (BMI ≥ 30 kg/m^2^) was significantly higher in ART-GDM group compared to non-GDM groups (ART and SC) (*p* < 0.001). The rate of previous history of GDM was significantly higher in SC-GDM group. Women in ART-GDM group had significantly higher rates of previous history of PCOS than the other groups (*p* < 0.001). The rate of maternal age of ≥35 years was significantly higher in GDM groups (ART-GDM and SC-GDM) than the SC group (*p* < 0.001). Together, a higher number of high-risk women were observed in ART-GDM. Gestational age at delivery was significantly lower among ART-GDM subjects than non-GDM groups (i.e. ART and SC groups). Gestational weight gain was significantly higher in non-GDM groups (i.e. ART and SC groups) than ART-GDM and SC-GDM groups. There was no significant difference between SC and ART in terms of GWG, gestational age at GDM screening and pre-pregnancy BMI (*P* > 0.05). There was no significant difference between SC-GDM and ART-GDM in maternal age, gestational age at delivery, pre-gravid BMI nor GWG (P > 0.05). Concurrent dietary management and insulin administration was required in 39 (25.7%) of women of SC-GDM group vs. 57 (41.8%) of women of ART-GDM group (*P* < 0.004). The rate of insulin therapy was significantly higher in ART-GDM subjects than SC-GDM individuals.

**Table 1:** Baseline characteristics of the study participants

Variables	ART (*n* = 125)	ART-GDM (*n* = 135)	SC-GDM (*n* = 152)	SC (*n* = 162)	*P*-Value
Maternal age (yr.) (Mean ± SD)	30.28 ± 4.93	32.11 ± 4.95	31.63 ± 5.49	28.81 ± 7.43	< 0.001^*^
Gestational age at GDM screening (wk.)	25.6 ± 3.5	22.7 ± 6.9	24.9 ± 5.6	24.6 ± 3.1	0< 001^a^
Gestational age at delivery (wk.) (Mean ± SD)	38.40 ± 1.15	37.78 ± 1.52	38.34 ± 1.47	39.05 ± 1.14	< 0.001^b^
Gravidity, n (%)					
1	97 (77.6)	80 (59.3)	53 (34.9)	85 (52.5)	< 0.001
≥ 2	28 (22.4)	55 (40.7)	99 (65.1)	77 (47.5)	
Parity, n (%)					
0	113 (90.4)	116 (85.9)	63 (41.4)	98 (60.5)	< 0.001
≥ 1	12 (9.6)	19 (14.1)	89 (58.6)	64 (39.5)	
Pre-gravid BMI (kg/m^2^) (Mean ± SD)	24.49 ± 3.95	27.38 ± 3.93	26.07 ± 4.93	24.14 ± 4.35	< 0.001^c^
Gestational weight gain (kg) (Mean ± SD)	15.03 ± 6.05	10.93 ± 5.09	11.57 ± 5.52	14.42 ± 6.12	< 0.001^d^
Previous history of PCOS, n (%)	10(8)	34 (25.2)	7 (7.3)	6(3.7)	< 0.001^e^
Maternal age ≥ 35 years	25(20)	38(28.2)	49(32.2)	22(13.7)	0.001 ^f^
Pre-gravid obesity (BMI ≥ 30 kg/m^2^), n (%)	14(11.4)	33(24.6)	25(16.9)	17(10.6)	0.004^g^
Previous history of GDM, n (%)	1(0.8)	4 (3)	21 (13.8)	1(0.6)	< 0.001^h^

*GDM* gestational diabetes mellitus, *SC* spontaneous conception, *ART* assisted reproductive technology. All of *P*-values for quantitative variables were determined by post-hoc analysis (LSD)

*ART vs. ART-GDM (*P* = 0.01), ART-GDM vs. SC-GDM (*P* = 0.5), ART -GDM vs. SC (*P* < 0.001), ART vs. SC (*P* = 0.03), SC-GDM vs. SC (*P* < 0.001), ART vs. SC-GDM (*P* = 0.06)

^a^ART vs. ART-GDM (*P* < 0.001). ART-GDM vs. SC -GDM (*P* < 0.001), ART -GDM vs. SC (*P* < 0.001), ART vs. SC (*P* = 0.1), SC -GDM vs. SC (*P* = 0.6), ART vs. SC -GDM (*P* = 0.2)

^b^ART vs. ART-GDM (*P* = 0.01), ART-GDM vs. SC –GDM = (*P* = 0.5), ART -GDM vs. SC (*P* < 0.001), ART vs. SC (*P* = 0.03), SC -GDM vs. SC (*P* < 0.001), ART vs. SC- GDM (*P* = 0.06)

^c^ART vs. ART-GDM (*P* < 0.001) ART-GDM vs. SC- GDM (*P* = 0.1), ART -GDM vs. SC (*P* < 0.001), ART vs. SC (*P* = 0.5), SC- GDM vs. SC (*P* < 0.001), ART vs. SC -GDM (*P* = 0.01)

^d^ART vs. ART-GDM (*P* < 0.001), ART-GDM vs. SC- GDM (*P* = 0.3), ART -GDM vs. SC (*P* < 0.001), ART vs. SC (*P* = 0.4), SC –GDM vs. SC (*P* < 0.001), ART vs. SC- GDM (*P* < 0.001)

^e^ART vs. ART-GDM (*P* < 0.001). ART-GDM vs. SC -GDM (*P* < 0.001), ART -GDM vs. SC (*P* < 0.001), ART vs. SC (*P* = 0.6), SC -GDM vs. SC (*P* = 0.1), ART vs. SC -GDM (*P* = 0.2)

^f^ART vs. ART-GDM (*P* = 0.126), ART-GDM vs. SC-GDM (*P* = 0.452), ART -GDM vs. SC (*P* = 0.002), ART vs. SC (*P* = 0.152), SC-GDM vs. SC (*P* < 0.001), ART vs. SC-GDM (*P* = 0.022)

^g^ART vs. ART-GDM (*P* = 0.006), ART-GDM vs. SC-GDM (*P* = 0.109), ART -GDM vs. SC (*P* = 0.001), ART vs. SC (*P* = 0.826), SC-GDM vs. SC (*P* = 105), ART vs. SC-GDM (*P* = 0.198)

^h^ART vs. ART-GDM (*P* = 0.205), ART-GDM vs. SC-GDM (*P* = 0.001), ART -GDM vs. SC (*P* = 0.118), ART vs. SC (*P* = 0.854), SC-GDM vs. SC (*P* < 0.001), ART vs. SC-GDM (*P* = 0.001)

### Risk of obstetric, perinatal and neonatal complications

Table 2 presents the obstetric outcomes observed in the four groups of the present study, along with the risk of each outcome relative to that in women of the SC group. Logistic regression showed that the risk of PIH was increased in the ART [adjusted odds ratio (aOR) 3.46, 95% confidence interval (CI), 3.46 (1.02, 11.68)], ART-GDM [aOR: 7.04, 95% CI: (2.24–22.15)] and GDM [aOR: 4.12, 95% CI: (1.31–12.89)] groups in comparison to the SC group. The ART [aOR: 5.29, 95% CI: (1.03–27.09)] and ART-GDM [aOR: 7.78, 95% CI: (1.62–37.47)] singleton pregnancies had higher risk of preeclampsia in comparison to SC pregnancies. It was found that the risk of APH was significantly increased only in ART-GDM group in comparison to SC group [aOR: 3.46, 95% CI: (1.28–9.33)]. Moreover, the risk of emergency CS was significantly higher in GDM groups (ART-GDM and SC-GDM) compared to the SC group.

**Table 2:** Risk of obstetrics, perinatal and neonatal complications among study groups

Variables	ART (*n* = 125)	ART-GDM (*n* = 135)	SC-GDM (*n* = 152)	SC (*n* = 162)
Pregnancy-induced hypertension (PIH), n (%)	10 (8.0)	25 (19.1)	16 (10.7)	4 (2.5)
OR (95% CI)	3.41 (1.04–11.15)*	9.26 (3.13–27.36)*	4.69 (1.53–14.36)*	Ref. group
Adjusted OR (95% CI)	3.46 (1.02–11.68)*	7.04 (2.24–22.15)*	4.12 (1.31–12.89)*	Ref. group
Preeclampsia, n (%)	7 (5.6)	15 (11.5)	7 (4.7)	2 (1.2)
OR (95% CI)	4.72 (0.96–23.11)	10.28 (2.31–45.83)*	3.89 (0.80–19.04)	Ref. group
Adjusted OR (95% CI)	5.29(1.03–27.09)*	7.78(1.62-37.47)*	2.80 (0.56–14.10)	Ref. group
Antepartum hemorrhage (APH), n (%)	11 (8.8)	21 (16.0)	11 (7.3)	6 (3.7)
OR (95% CI)	2.49 (0.90–6.94)	4.93 (1.93–12.62)*	2.04 (0.74–5.67)	Ref. group
Adjusted OR (95% CI)	2.24 (0.78–6.40)	3.46 (1.28–9.33)*	1.80 (0.63–5.14)	Ref. group
Emergency Cesarean Section, n (%)	29 (23.2)	51 (39.2)	32 (21.8)	24 (15.1)
OR (95% CI)	1.70 (0.93–3.10)	3.63 (2.08–6.35)*	1.57 (0.87–2.81)	Ref. group
Adjusted OR (95% CI)	1.24 (0.67–2.32)	2.64 (1.43–4.88)*	2.01 (1.09–3.73)*	Ref. group

*GDM* gestational diabetes mellitus, *SC* spontaneous conception, *ART* assisted reproductive technology, *PIH* pregnancy induced hypertension

*OR* Crude Odds Ratio, *CI* Confidence Interval

OR were adjusted for maternal age, parity and pre-pregnancy BMI

Ref. means reference group

*P < 0.05 was considered significant

Perinatal and neonatal outcomes are shown in Table 3. The risk of preterm birth, LBW and neonatal hypoglycemia in the ART-GDM group were increased in comparison to the SC group [(aOR: 3.89, 95% CI: (1.51–10.10)), (aOR: 3.11, 95% CI: (1.04–9.30)), and (aOR: 4.91, 95% CI: (1.50–16.07)), respectively]. Furthermore, the SC-GDM pregnancies compared to the SC pregnancies, had a significantly higher risk of developing LGA [aOR: 5.20, 95% CI: (1.07–25.20)]. In addition, the ART-GDM [aOR: 4.36, 95% CI: (1.82–10.45)] and ART [aOR: 2.53, 95% CI: (1.05–6.09)] pregnancies indicated a higher risk of NICU admission in comparison to SC pregnancies.

**Table 3:** Risk of perinatal and neonatal complications among study groups

Variables	ART (*n* = 125)	ART-GDM (*n* = 135)	SC-GDM (*n* = 152)	SC (*n* = 162)
Preterm Birth (PTB), n (%)	9 (7.2)	23 (17.3)	13 (8.7)	7 (4.3)
OR (95% CI)	1.71 (0.62–4.72)	4.60 (1.90–11.10)*	2.09 (0.81–5.38)	Ref. group
Adjusted OR (95% CI)	1.53 (0.54–4.35)	3.89 (1.51–10.10)*	2.13 (0.81–5.63)	Ref. group
IUGR n (%)	12 (9.6)	14 (10.8)	10 (6.7)	5 (3.1)
OR (95% CI)	3.27 (1.12–9.55)	3.72 (1.30–10.61)	2.20 (0.73–6.59)	Ref. group
Adjusted OR (95% CI)	2.63 (0.88–7.89)	2.92 (0.96–8.93)	2.57 (0.83–7.93)	Ref. group
Small for gestational age (SGA), n (%)	18 (14.4)	20 (15.3)	9 (6.0)	18 (11.2)
OR (95% CI)	1.34 (0.66–2.69)	1.43 (0.72–2.83)	0.51 (0.22–1.17)	Ref. group
Adjusted OR (95% CI)	1.14 (0.55–2.37)	1.34 (0.62–2.87)	0.62 (0.27–1.46)	Ref. group
Large for gestational age (LGA), n (%)	3 (2.4)	7 (5.3)	9 (6.0)	2 (1.2)
OR (95% CI)	1.95 (0.32–11.88)	4.49 (0.92–21.98)	5.07 (1.08–23.88)*	Ref. group
Adjusted OR (95% CI)	2.19 (0.34–14.03)	5.08 (0.92–28.15)	5.20(1.07–25.20)*	Ref. group
Macrosomia n (%)	3 (2.4)	4 (3.1)	7 (4.7)	4 (2.5)
OR (95% CI)	0.97 (0.21–4.39)	1.25 (0.31–5.08)	1.92 (0.55–6.70)	Ref. group
Adjusted OR (95% CI)	1.01 (0.21–4.90)	1.21 (0.26–5.66)	1.88 (0.51–6.83)	Ref. group
Low birth weight (LBW), n (%)	10 (8.0)	16 (12.2)	8 (5.3)	5 (3.1)
OR (95% CI)	2.7 (0.90–8.15)	4.34 (1.55–12.19)*	1.76 (0.56–5.50)	Ref. group
Adjusted OR (95% CI)	1.99 (0.65–6.08)	3.11 (1.04–9.30)*	2.23 (0.70–7.14)	Ref. group
NICU admission n (%)	18 (14.4)	27 (20.8)	12 (8.0)	9 (5.6)
OR (95% CI)	2.82 (1.22–6.52)*	4.40 (1.99–9.74)*	1.46 (0.60–3.57)	Ref. group
Adjusted OR (95% CI)	2.53 (1.05–6.09)*	4.36 (1.82–10.45)*	1.59 (0.64–3.97)	Ref. group
Respiratory distress n (%)	7 (5.6)	15 (11.5)	12 (8.1)	15 (9.3)
OR (95% CI)	0.58 (0.23–1.46)	1.27 (0.60–2.70)	0.85 (0.39–1.89)	Ref. group
Adjusted OR (95% CI)	0.48 (0.18–1.26)	1.02 (0.44–2.34)	0.96(0.42–2.18)	Ref. group
Neonatal hypoglycemia, n (%)	4(3.2)	15(11.5)	5(3.3)	5(3.1)
OR (95% CI)	1.02(0.27–3.90)	4.04(1.43–11.45)*	1.07(0.30-3.77)	Ref. group
Adjusted OR (95% CI)	1.17(0.29–4.72)	4.91(1.50–16.07)*	0.94(0.24-3.70)	Ref. group
Perinatal mortality, n (%)	0	5(3.7)	2(1.3)	1(0.6)
OR (95% CI)	1	6.19(0.71–53.66)	2.15(0.19–23.92)	Ref. group
Adjusted OR (95% CI)	1	5.56(0.55–56.65)	2.23(0.19–25.87)	Ref. group

*GDM* gestational diabetes mellitus, *SC* spontaneous conception, *ART* assisted reproductive technology, *PIH* pregnancy induced hypertension, *IUGR* Intrauterine growth restriction*; NICU* neonatal intensive care unit

*OR* Crude Odds Ratio, *CI* Confidence Interval

ORs were adjusted for maternal age, parity and pre-pregnancy BMI

Ref. means reference group

*P < 0.05 was considered significant

The risk of other evaluated perinatal complications (e.g. SGA, respiratory distress, macrosomia and IUGR) did not show any differences among the study groups. Perinatal mortality was observed in 5 (3.7%) women of the ART-GDM, 2 (1.3%) women of the SC-GDM and 1 (0.6%) woman of the SC groups (*P* = 0.052). Neonatal hypoglycemia was observed in 5 (3.3%) neonates of the SC-GDM and 15 (11.5%) neonates of the ART-GDM groups (*P* < 0.004). Apgar scores < 7 at 5 min were observed in 2 (1.5%) neonates of the ART-GDM and 6 (4.1%) neonates of the SC-GDM groups (*P* < 0.004). Apgar scores < 7 at 5 min were not observed in the SC group.

Table 4 shows the pairwise comparison of adverse pregnancy outcomes in different groups. The results showed that the ART-GDM group had higher risks of emergency CS, PTB and neonatal hypoglycemia in comparison to the ART group. Additionally, ART-GDM group had higher risks of preeclampsia, NICU admission and neonatal hypoglycemia compared to the SC-GDM group.

**Table 4:** Pairwise comparisons of obstetric and perinatal outcomes among study population

Variables	ART-GDM vs. ART	ART-GDM vs. SC- GDM	ART vs. SC-GDM
Obstetrics outcomes			
PIH, OR adjusted (95% CI)	2.09 (0.91–4.81)	1.83 (0.79–4.27)	0.79 (0.30–2.11)
Preeclampsia, OR adjusted (95% CI)	1.45 (0.53–3.99)	3.31(1.03–10.64) *	1.56 (0.41–5.89)
Antepartum hemorrhage, OR adjusted (95% CI)	1.57(0.69–3.57)	1.88(0.74–4.79)	1.54(0.53–4.50)
Emergency CS, OR adjusted (95% CI)	1.95 (1.08–3.50) *	1.27(0.68–2.39)	1.43 (0.74–2.77)
Perinatal outcomes			
Preterm birth, OR adjusted (95% CI)	2.46(1.04–5.85) *	1.60(0.67–3.84)	0.84 (0.30–2.38)
IUGR, OR adjusted (95% CI)	1.04(0.43–2.52)	0.96(0.34–2.69)	1.05(0.39–2.86)
Small for gestational age, OR adjusted (95% CI)	1.05(0.50–2.22)	1.82(0.70–4.73)	1.89(0.74–4.84)
Large for gestational age, OR adjusted (95% CI)	2.54(0.58–11.10)	1.13(0.33–3.87)	0.55(0.12–2.53)
Macrosomia, OR adjusted (95% CI)	1.39(0.27–7.14)	0.71(0.16–3.22)	0.63(0.13–3.01)
Low birth weight, OR adjusted (95% CI)	1.53(0.62–3.74)	1.48(0.52–4.18)	1.14(0.38–3.41)
NICU admission, OR adjusted (95% CI)	1.91(0.93–3.93)	2.94(1.23–7.05) *	1.42(0.57–3.53)
Respiratory distress, OR adjusted (95% CI)	2.29(0.84–6.23)	1.09(0.42–2.80)	0.44(0.15–1.28)
Neonatal hypoglycemia, OR adjusted (95% CI)	4.68(1.04–15.65) *	6.53(1.77–24.16) *	2.02(0.36–11.43)
Perinatal mortality, OR adjusted (95% CI)	1	3.07(0.41–22.77)	1

*GDM* gestational diabetes mellitus, *SC* spontaneous conception, *ART* assisted reproductive technology, *PIH* pregnancy induced hypertension, *IUGR* Intrauterine growth restriction, *NICU* neonatal intensive care unit

*OR* Crude Odds Ratio, *CI* Confidence Interval

ORs were adjusted for age, parity and pre-pregnancy body mass index

*P < 0.05 was considered significant

As it is shown in Table 5, ART subjects compared to non-ART ones, had higher risks of PIH, preeclampsia, APH, NICU admission, and neonatal hypoglycemia. Moreover, GDM individuals compared to non-GDM ones, had higher risks of PIH, emergency CS, preterm birth, LGA and neonatal hypoglycemia.

**Table 5:** Interaction between ART and GDM in obstetrics and perinatal outcomes

Variables	ART vs. Non-ART	GDM vs. Non-GDM
Obstetrics outcomes		
PIH, adjusted OR (95% CI)	2.09 (1.05–4.14)*	2.68 (1.38–5.20)*
Preeclampsia, adjusted OR (95% CI)	3.40 (1.34–8.66)*	1.77 (0.76–4.12)
Antepartum hemorrhage, adjusted OR (95% CI)	2.04(1.01–4.11)*	1.64(0.86-3.13)
Emergency CS, adjusted OR (95% CI)	1.28 (0.82–1.99)	2.07 (1.35–3.17)*
Perinatal outcomes		
Preterm birth, adjusted OR (95% CI)	1.71(0.87–3.36)	2.36(1.24–4.49)*
IUGR, adjusted OR (95% CI)	1.65(0.79–3.44)	1.53(0.77–3.01)
Small for gestational age, adjusted OR (95% CI)	1.47(0.82–2.64)	0.90(0.52–1.55)
Large for gestational age, adjusted OR (95% CI)	1.21(0.43–3.38)	3.45(1.20–9.96)*
Macrosomia, adjusted OR (95% CI)	0.77(0.26–2.33)	1.5(0.57–4.32)
Low birth weight, adjusted OR (95% CI)	1.63(0.77–3.46)	1.78(0.88–3.60)
NICU admission, adjusted OR (95% CI)	2.64(1.41–4.94)*	1.68(0.95-2.94)
Respiratory distress, adjusted OR (95% CI)	0.73(0.38–1.42)	1.35(0.73–2.50)
Neonatal hypoglycemia, adjusted OR (95% CI)	2.86(1.12–7.30)*	2.36(1.00-5.57)*
Perinatal mortality, adjusted OR (95% CI)	1.59 (0.29–8.70)	6.97(0.81–59.58)

*GDM* gestational diabetes mellitus, *SC* spontaneous conception, *ART* assisted reproductive technology, *PIH* pregnancy induced hypertension, *IUGR* Intrauterine growth restriction, *NICU* neonatal intensive care unit

*OR* Crude Odds Ratio, *CI* Confidence Interval

ORs were adjusted for age, parity and pre-pregnancy body mass index

*P < 0.05 was considered significant

## Discussion

In this prospective cohort study, pregnant women conceived via ART were compared with women with SC, in terms of obstetric and perinatal outcomes of GDM. Three main findings were as follows: Shorter durations of gestation and lower GWG were observed in GDM groups (ART-GDM and SC-GDM) compared to SC; ART-GDM pregnancies had higher risk of PIH, preeclampsia, APH and emergency CS compared to SC; Also, ART-GDM group had higher risk of perinatal and neonatal outcomes with respect to PTB, LBW, NICU admission, and neonatal hypoglycemia compared to SC. The risk of LGA was significantly higher in the SC-GDM group compared to SC group.

In the current study, mothers in SC group were significantly younger than the other groups. Moreover, rate of pre-gravid BMI in GDM groups (ART-GDM and SC-GDM) was significantly higher than those of non-GDM groups (ART and SC). Conversely, GDM groups had significantly lower total GWG compared to non-GDM groups, which was potentially due to more strict weight and diet management during pregnancy, as well as lower gestational age at delivery. Previous studies found decreasing total GWG with increasing pre-gravid BMI, but higher rate of mothers with extreme GWG in overweight group [24–26]. However, since, in the present study, GWG was not evaluated based on BMI as explained by Institute of Medicine (IOM) recommendation, we do not have enough evidence to fully discuss our results [27].

According to the present study, over 85% of ART subjects (ART and ART-GDM) were nulliparous. Insulin administration in ART-GDM group (41.8%) was significantly higher than that of SC-GDM group (25.7%). In a recent study, insulin was given to 28.1% of women with GDM [17]. Insulin resistance is considered the main etiology of GDM [28]. It was shown that infertile women especially those with PCOS, exhibit markedly higher levels of insulin resistance and oxidative stress [29, 30].

One of our main findings was significantly increased risks of obstetric outcomes including PIH, preeclampsia, APH and emergency CS in ART-GDM group as compared to SC. Both ART groups had higher risks of PIH and preeclampsia; also, both GDM groups had higher risks of PIH and emergency CS compared to SC. Although little evidence in available concerning the adverse pregnancy outcomes of GDM following ART treatment, there are several reviews and meta-analyses supporting the hypotheses that singleton pregnancy after ART poses higher risks of obstetric outcomes when compared with natural conception [7, 13, 31, 32]. Our data showed that PIH rate in GDM-ART women was higher (19.1%) than women in ART (8%), SC-GDM (10.7%) and SC (2.5%) groups. Previously, Szymanska et al. showed higher rates of preeclampsia in IVF-GDM group (36 women) compared to non-IVF women with GDM (137 women) (8.3% vs. 3.6%, respectively) [21]. Moreover, Ashrafi et al. reported higher incidence of PIH in IVF/ICSI group compared to SC (21 vs. 7%, respectively) [9]. In a similar way, Tandberg et al. [33] reported that ART elevates the risk of preeclampsia and it might be even worsened by parity. Nevertheless, Watanabe et al. [34] indicated that the relationship between IVF and preeclampsia might be confounded by residual unmeasured factors.

Furthermore, our findings demonstrated that women with GDM (ART-GDM and SC-GDM) had a higher risk of emergency CS. Consistently, previous evidence showed that GDM is positively correlated with emergency CS, particularly among nulliparous GDM women and LGA infants [35, 36]. Moreover, increased risk of obstetric hemorrhage in singleton birth after ART and GDM pregnancies was reported [37, 38]. Though it is not fully understood, several factors including specific infertility and ART characteristics, maternal factors, and metabolic disturbance or a combination of these factors, have been linked to pregnancy-related complications in ART-GDM subjects [13, 21, 39–41]. During fertilization and preimplantation development, these factors may contribute to intracellular metabolic and epigenetic modifications which may eventually induce deleterious consequences to prenatal development and post-natal growth [42].

Intrestingly, we observeed that ART-GDM pregnancies had significantly increased risks of adverse perinatal outcomes including PTB, LBW and NICU admission compared to naturally conceived ones after adjustment for potential confounding factors. Furthermore, both GDM groups had higher risks of LGA compared to the reference group and this difference was statistically significant when comparing SC-GDM group and the reference group. GDM is considered an important risk factor for fetal macrosomia and LGA newborn especially in women with untreated or undetected GDM [43]. Our results showed that compared to spontaneously conceived pregnancies, ART-GDM pregnancies are delivered 2 weeks earlier and those conceived by ART and SC-GDM are delivered 1 week earlier. Lower rates of macrosomia, LGA and GWG in ART-GDM group may be legitimized by shorter duration of pregnancy and higher rate of PTB.

The present data showed higher rates of PTB (17.3%) and LBW (12.2%) in ART-GDM compared to the other three groups. The worldwide prevalence of PTB and LBW among IVF/ICSI pregnancies was found to be 10.9 and 8.7%, respectively [13]. In a recent meta-analysis of cohort studies, Cavoretto et al. showed high incidence of spontaneous PTB in singleton IVF/ICSI pregnancies compared to those conceived naturally (10.1 vs. 5.5%, respectively); odds ratio (OR), 1.75; 95% CI, 1.50–2.03) [44]. Wisborg et al. [45] found that the risk of PTB in subfertile and fertile subjects is quite similar, and the risk of PTB in IVF/ICSI subjects is related to the ART treatment. They found no association between IVF/ICSI and the risk of LBW or NICU admission rate. It was indicated that genetic and environmental factors, medical conditions of mother or fetus, ART methods, behavior and socioeconomic elements, and iatrogenic prematurity may contribute to PTB in ART [46]. Our findings showed an association between ART-GDM and elevated odds of PTB and LBW. Nonetheless, such associations were not observed in ART groups. This may be partially explained by dissociation between ART and GDM-ART in the current study as previous studies did not discriminate ART from ART-GDM and/or other co-comorbidities.

Our results demonstrated that the neonates of ART groups, particularly ART-GDM group, had higher risks of NICU admission. Furthermore, higher rates of PTB, IUGR, SGA, LBW and emergency CS were observed in ART group, though their associations with ART were not statistically significant. This may be due to small sample size of our study. In addition, better healthcare may explain higher rates of NICU admission in ART group.

In the current study, a higher rate of perinatal mortality (3.7%) was observed in ART-GDM group. Furthermore, a higher rate of neonatal hypoglycemia was observed in ART-GDM groups (11.5%) compared to SC-GDM (3.3%). Insulin requirement rate was higher in ART-GDM group possibly due to higher insulin resistance in ART-GDM group.

Our data indicated that both ART versus non-ART pregnancies and GDM versus non-GDM pregnancies are closely related to higher risks of pregnancy- related complications. Of note, ART-GDM pregnancies were associated with higher risks of emergency CS, PTB, and neonatal hypoglycemia compared to ART pregnancies alone. The current study confirmed that ART-GDM pregnancy was related to higher risks of adverse pregnancy outcomes compared to both non-GDM ART and SC-GDM. In this research, there were no significant differences between SC-GDM and ART-GDM in terms of maternal age, gestational age at delivery, pre-pregnancy BMI and gestational weight gain, albeit obstetric and perinatal outcomes were more prevalent in ART-GDM compared to SC-GDM which might be caused by ART treatment. However, literature lacks sufficient evidence to show possible correlations between GDM and ART treatment and the underlying mechanisms. Pinborg et al. in a systematic review, discussed parameters which affect perinatal risks in ART singletons and categorized them in four major groups namely, subfertility per se, controlled ovarian hyperstimulation, laboratory procedures and number of embryos transferred [31]. Therefore, recent strategies such as milder ovarian stimulation, single embryo transfer, improvements of lab techniques and utilization of better culture media have been taken to overcome such problems.

So far, there has been no prospective cohort study evaluating obstetric and perinatal outcomes of GDM following ART. To the best of our knowledge, the present research is the first observational cohort study conducted in a GDM-ART group. Nevertheless, the present study had a small sample size. Therefore, it is urgent to perform such an experiment in a larger population. Further research is needed to confirm increased risk of obstetric and perinatal complications after ART-GDM and also determine which aspects of ART induce adverse pregnancy outcomes following GDM and how this risk can be minimized.

## Conclusion

In conclusion, singletons pregnancies conceived by ART-GDM have higher risk of adverse obstetric and perinatal outcomes compared to SC (for PTB, LBW, NICU admission and neonatal hypoglycemia). In addition, ART-GDM has higher risk of emergency CS, PTB, and neonatal hypoglycemia compared to ART alone. SC-GDM pregnancies have higher risk of LGA compared to SC.

## Abbreviations

ADA/IAPDSG: American diabetes association/ International association of diabetes and pregnancy study groups
ANOVA: Analysis of variance
aOR: Adjusted odds ratio
ART: Assisted reproductive technology
BSP: Blood sugar profile
GDM: Gestational diabetes mellitus
GWG: Gestational weight gain
IOM: Institute of medicine
IUGR: Intrauterine growth restriction
IVF/ICSI: In vitro fertilization/ Intra-cytoplasmic sperm injection
LBW: Low birth weight
LGA: Large for gestational age
NICU: Neonatal intensive care unit
OGTT: Oral glucose tolerance test
PCOS: Polycystic ovary syndrome
PIH: Pregnancy induced hypertension
SC: Spontaneous conception
SGA: Small for gestational age
